# The Role of Artificial Intelligence in Pediatric Intensive Care: A Systematic Review

**DOI:** 10.7759/cureus.80142

**Published:** 2025-03-06

**Authors:** Almontasir Belah Alsadig Abdalwahab Abdallah, Sally Ibrahim Hafez Sadaka, Elryah I Ali, Saadalnour Abusail Mustafa Bilal, Mohammad Omar Abdelrahman, Fatima Bashir Fakiali Mohammed, Samah Dafallah Nimir Ahmed, Nuha Elrayah Abdelrahim Saeed

**Affiliations:** 1 Department of Pediatrics, Maternity and Children Hospital, Najran, SAU; 2 Department of Pediatrics, Armed Forced Hospital Najran, Najran, SAU; 3 Department of Medical Laboratory Technology, College of Applied Medical Sciences, Northern Border University, Arar, SAU; 4 Department of Clinical Laboratory Science, College of Applied Medical Sciences, Najran University, Najran, SAU; 5 Department of Obstetrics and Gynecology, Maternity and Children Hospital, Najran, SAU; 6 Department of Pediatrics, Dawadmi General Hospital, Al Duwadimi, SAU; 7 Department of Pediatrics, Southend University Hospital NHS Foundation Trust, Southend-on-Sea, GBR; 8 Department of Biochemistry, University of Khartoum, Khartoum, SDN; 9 Department of Pediatrics, Al Enjaz Medical Center, Riyadh, SAU

**Keywords:** artificial intelligence, health outcome, pediatric intensive care unit, pediatrics, picu

## Abstract

Pediatric intensive care units (PICUs) could transform due to artificial intelligence (AI), which could improve patient outcomes, increase diagnostic accuracy, and streamline repetitive procedures. The goal of this systematic review was to outline how AI can be used to enhance any health outcomes in pediatric intensive care. We searched four databases (PubMed, Scopus, Web of Science, and IEEE Xplore) for relevant studies using a predefined systematic search. We found 267 studies in these four databases. The studies were first screened to remove the duplicates and then screened by titles to remove irrelevant studies. The studies were further screened based on inclusion and exclusion criteria, in which 32 studies were found suitable for inclusion in this study. The studies were assessed for risk of bias using the Prediction Model Risk Of Bias Assessment Tool (PROBAST) tool. After AI was implemented, almost 22% (n = 7) of studies showed an immediate effect and enhanced health outcomes. A small number of studies involved AI implementation in actual PICUs, while the majority focused on experimental testing. AI models outperformed conventional clinical modalities among the remaining 78% (n = 25) and might have indirectly impacted patient outcomes. Significant variation in metrics and standardization was found when health outcomes were quantitatively assessed using statistical measures, including specificity (38%; n = 12) and area under the receiver operating characteristic curve (AUROC) (56%; n = 18). There are not sufficient studies showing that AI has significantly enhanced pediatric critical care patients' health outcomes. To evaluate AI's impact, more prospective, experimental research is required, utilizing verified outcome measures, defined metrics, and established application frameworks.

## Introduction and background

A significant increase in research investigations, especially in the area of critical care medicine, indicates that artificial intelligence (AI), driven by its potential to improve patient outcomes and support professional decisions, is poised to profoundly revolutionize medicine [1]. The increasing number of clinical studies on AI-related topics reflects this growth. Furthermore, generative AI, particularly large language models (LLMs), such as ChatGPT, is gaining traction in healthcare settings, providing decision support and assisting with documentation and communication tasks. Early perceptions and experiences of ChatGPT in pediatric critical care among healthcare professionals highlight both its potential and challenges [2].

AI may help clinicians with diagnosis, prognostics, and treatment in pediatric intensive critical care to improve patient outcomes, despite the difficulties in research brought on by ethical and practical concerns [3]. But there is still a long way to go before the advances in AI research are translated into real clinical advantages and used at the patient's bedside. Currently, less than 2% of AI models progress beyond the prototype phase, limiting their real-world impact [4]. Bias is a major concern for AI models, and the majority of patients in these studies are adults, making it uncertain how these findings apply to pediatric populations. This is crucial because pediatric healthcare encompasses a wide range of developmental phases, from infancy to puberty, leading to a highly heterogeneous patient population with notable anatomical and physiological variances [5]. Adults and children have significant differences in disease incidence, appearance, outcomes, and prognosis, which results in different clinical needs, evaluations, and treatment strategies for each group [6]. Thus, there is a critical need to bridge this gap by developing AI models specifically tailored to pediatric care.

A number of guidelines have put forth frameworks for the ethical design, behavior, reporting, and early-stage assessment of AI research in an effort to close the gap between conventional quantitative measurements and the application of AI at the bedside [7]. One such framework, the "Recommendations for the Safe Inclusion of Pediatric Data in Artificial Intelligence and Machine Learning Research" (ACCEPT-AI), was put forth to create secure and reliable AI applications tailored for pediatrics, taking into account the anatomical and physiological distinctions between adults and children as well as the variability in developmental phases within the pediatric population [8]. This framework highlights the need for pediatric-specific AI research and lists the technology and research factors that are crucial for creating reliable and safe AI models for this demographic.

Despite these efforts, a clear research gap remains regarding the real-world effectiveness of AI in pediatric intensive care. While previous reviews have addressed AI's role in adult populations and general healthcare, fewer studies have systematically evaluated its impact in pediatric intensive care units (PICUs). Determining how ready AI technology is for practical application in critically ill pediatric patients is essential because it may help medical professionals with diagnosis, prognostics, and therapy across pediatric care, as well as improve patient outcomes. With this knowledge, we will be able to recognize possible obstacles and proceed with clinical implementation. Therefore, the goal of this systematic review is to evaluate the body of research on the current application of AI in PICUs, as well as the potential for bias. Additionally, this review aims to highlight key challenges in AI adoption, including data scarcity, model validation, and the need for standardized evaluation metrics. We also assessed the various AI experiments' preparedness for clinical application.

## Review

Methodology

Review Protocol

This systematic review was carried out in accordance with the Preferred Reporting Items for Systematic Reviews and Meta-Analyses (PRISMA) 2020 guidelines [9]. An established procedure that described the objectives, inclusion/exclusion criteria, and analysis methods was established prior to data collection. The exploratory nature of the evaluation prevents the protocol from being listed in a public registry.

Search Strategy

A systematic search was conducted in PubMed, Scopus, Web of Science, and IEEE Xplore to identify relevant studies on the role of AI in pediatric intensive care. The search strategy incorporated a combination of keywords and Medical Subject Headings (MeSH) terms related to AI and critical care. Additionally, the reference lists of included studies were screened to identify any additional relevant articles. The detailed search strings for each database are given in Table 1.

**Table 1:** Search strategy for each database

Database	Search string
PubMed	("Artificial Intelligence"[MeSH] OR "Machine Learning"[MeSH] OR "Deep Learning"[MeSH] OR "Neural Networks, Computer"[MeSH] OR "Artificial Intelligence" OR "Machine Learning" OR "Deep Learning" OR "Neural Networks") AND ("Pediatric Intensive Care Units"[MeSH] OR "Pediatric Critical Care"[MeSH] OR "PICU" OR "Pediatric Intensive Care" OR "Critical Care" OR "Intensive Care Unit" OR "ICU")
Scopus	(TITLE-ABS-KEY("Artificial Intelligence" OR "Machine Learning" OR "Deep Learning" OR "Neural Networks")) AND (TITLE-ABS-KEY("Pediatric Intensive Care" OR "Pediatric Critical Care" OR "PICU" OR "Intensive Care Unit" OR "ICU"))
Web of Science	TS=("Artificial Intelligence" OR "Machine Learning" OR "Deep Learning" OR "Neural Networks") AND TS=("Pediatric Intensive Care" OR "Pediatric Critical Care" OR "PICU" OR "Intensive Care Unit" OR "ICU")
IEEE Xplore	("Artificial Intelligence" OR "Machine Learning" OR "Deep Learning" OR "Neural Networks") AND ("Pediatric Intensive Care" OR "Pediatric Critical Care" OR "PICU" OR "Intensive Care Unit" OR "ICU")

Eligibility Criteria

The eligibility criteria for this systematic review were determined using the PICOS (Population, Intervention, Comparison, Outcome, and Study Design) framework to ensure a structured and comprehensive selection of relevant studies. Population (P): Studies focusing on pediatric patients admitted to PICUs were included. Research involving neonatal or adult intensive care populations was excluded unless the results for pediatric patients were reported separately. Intervention (I): The review included studies where AI, machine learning (ML), or deep learning (DL) models were applied in PICU settings. AI applications could include, but were not limited to, predictive modeling for clinical deterioration, automated diagnosis, treatment decision support, and patient monitoring. Comparison (C): Studies with a comparator, such as conventional clinical scoring systems, physician assessments, or other AI-based models, were preferred. However, studies without a direct comparator were also considered if they reported AI performance metrics in a clinically relevant context. Outcomes (O): Included studies were required to report on at least one performance metric, such as accuracy, area under the receiver operating characteristic curve (AUROC), sensitivity, specificity, precision, or F-score. Studies that did not provide quantitative outcome measures or lacked sufficient methodological details were excluded. Study Design (S): Only peer-reviewed original research articles were included. Systematic reviews, narrative reviews, conference abstracts, case reports, editorials, and opinion pieces were excluded. Studies published in English were considered, and those in other languages were excluded unless a reliable English translation was available. We did not consider specific publication dates to ensure the inclusion of both foundational research and recent advancements, providing a comprehensive overview of AI's development and impact in pediatric intensive care.

Study Selection

Two independent reviewers (ABA and SM) screened titles and abstracts for eligibility. Full-text screening was conducted for potentially relevant studies. Disagreements were resolved through discussion or consultation with a third reviewer (FBFM). The PRISMA guidelines were followed for study selection and reporting.

Data Extraction and Synthesis

A standardized data extraction form was used to collect information on study characteristics (authors, year), AI methodology (type of algorithm, dataset size, feature selection), clinical application (diagnosis, monitoring, outcome prediction), and performance metrics (accuracy, AUROC, sensitivity, specificity, F-measure). Data were synthesized narratively due to heterogeneity in AI models and outcome reporting.

Risk of Bias Assessment

The risk of bias in the included studies was evaluated using the Prediction model Risk Of Bias Assessment Tool (PROBAST). This tool assesses bias across four domains: participants, predictors, outcome, and analysis. Two independent reviewers (ABA and SM) conducted the assessment, resolving disagreements through discussion. Each study was rated as having a low, high, or unclear risk of bias based on predefined criteria. The assessment focused on study design, participant selection, predictor definition, outcome measurement, and statistical analysis. Studies with poor methodological transparency or inadequate handling of missing data were flagged as high risk. The final ratings were compiled into a summary table for analysis.

Results

Search Results

**Figure 1:**
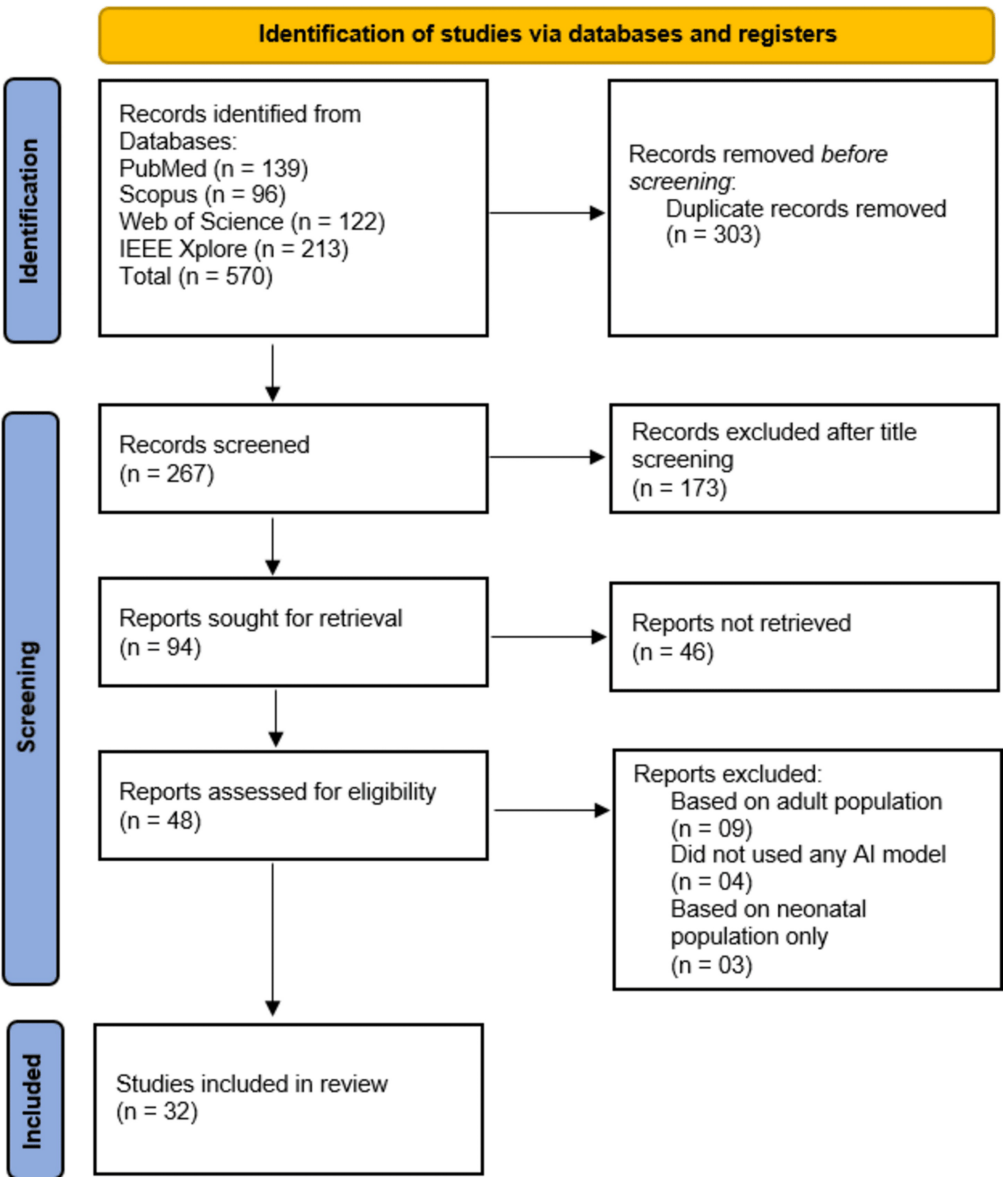
PRISMA flow diagram PRISMA: Preferred Reporting Items for Systematic Reviews and Meta-Analyses

A systematic search was conducted across PubMed, Scopus, Web of Science, and IEEE Xplore on 09-14 January 2025, which identified a total of 570 records. After the removal of 303 duplicate records, 267 unique records were screened based on titles and abstracts, leading to the exclusion of 173 studies for irrelevance. The full texts of 94 studies were sought for retrieval, but 46 were not accessible due to paywall restrictions. Among the 48 retrieved full-text studies, 16 were excluded - nine for focusing on adult populations, four for not utilizing AI models, and three for exclusively studying neonatal populations. Finally, 32 studies met the eligibility criteria and were included in this systematic review (Figure 1).

Characteristics of Included Studies

The systematic review included 32 studies that investigated the role of AI in pediatric intensive care. The study designs varied, with most being retrospective cohort studies, while some employed randomized controlled trials or prospective observational designs. Sample sizes ranged from 15 to 150,000 patients, reflecting the diversity in dataset availability and study scope. The primary objectives of these studies focused on predicting patient outcomes, diagnosing critical conditions, and optimizing treatment strategies using AI models. Various AI algorithms were utilized, including artificial neural networks (ANNs), support vector machines (SVMs), convolutional neural networks (CNNs), Bayesian models, and decision trees, among others. Most studies adopted supervised learning approaches, leveraging data from electronic health records (EHRs), clinical databases, and imaging techniques. The AI models were predominantly used for binary classification tasks, such as predicting mortality, sepsis, and neurological outcomes, while others focused on regression or multiclass classification. Cross-validation methods were inconsistently reported, with some studies employing fivefold or tenfold validation, while others did not specify their validation techniques. Overall, the included studies demonstrated the growing application of AI in early diagnosis, risk stratification, and clinical decision support within pediatric intensive care settings (Table 2).

**Table 2:** Characteristics of all included studies in this systematic review AI: artificial intelligence; ANN: artificial neural network; AODE: averaged one-dependence estimators; AUROC: area under the receiver operating characteristic curve; CNN: convolutional neural network; CPD: cerebral palsy detection; EHR: electronic health record; fMRI: functional magnetic resonance imaging; GB: gradient boosting; HER: health electronic record; ICU: intensive care unit; iROP: imaging and retinopathy of prematurity; k-NN: k-nearest neighbors; LOS: length of stay; LR: logistic regression; naïve Bayes: naïve Bayes classifier; NICU: neonatal intensive care unit; PICU: pediatric intensive care unit; PROBAST: prediction model risk of bias assessment tool; RF: random forest; SVM: support vector machine

Author and publishing year	Study design	Sample size	Objective	AI algorithm	Classification type	Data source	Learning type	Outcome	Cross-validation
Saria et al., (2010) [10]	Retrospective cohort study	138	Develop a technology that assesses a newborn's physiological state and forecasts the severity of their future disease.	PhysiScore	Regression	EHR	Supervised	Morbidity and mortality	Not reported
Saadah et al., (2014) [11]	Retrospective study	176	Determine which premature newborns may benefit from palivizumab prevention during nosocomial respiratory infection epidemics.	ANN	Binary	Research database	Supervised	Mortality, days of extra oxygen, and duration of ICU stay	Not reported
Matić et al., (2015) [12]	Retrospective study	53	Create an algorithm to measure the dynamics of background electroencephalograph in neonates suffering from hypoxia ischemic encephalopathy.	Least-square SVM	Multiclass	Polygraphic EEG method for video surveillance	Supervised	Neurodevelopmental outcome	Fivefold
Caparros-Gonzalez et al., (2018) [13]	Double-blind randomized controlled trial	1039	Describe how a music therapy intervention affects premature infants' heart rates, blood pressure, oxygen saturation, and breathing rates.	Regression tree	Regression	Observation and vitals	Supervised	Stress-related condition marked by modifications to the autonomic nervous system	Fivefold
He et al., (2018) [14]	Retrospective cohort study	28	Provide a paradigm for predicting cognitive impairments and outcomes in preterm infants based on resting state fMRI functioning connectome data.	ANN	Binary	Research database	Unsupervised	Cognitive outcome	10 fold
Podda et al., (2018) [15]	Retrospective cohort study	23747	Create a technique for assessing the survival of preterm as well as low birth weight infants to forecast their survival.	ANN	Binary	Research database	Supervised	Mortality prior to ICU discharge	Fivefold
Clark et al., (2019) [16]	Prospective and retrospective	401	Rapid whole-genome sequencing can be used to identify genetic disorders and forecast the probability of death and morbidity in children who are very sick.	Bayesian models, neural networks, decision trees, and natural language processing	Multiclass	EHR	Supervised	Morbidity and mortality	Not reported
Temko et al., (2011) [17]	Retrospective cross-sectional	17	Provide a multichannel and patient-independent method to identify seizures, which are frequently a sign of brain damage in infants.	SVM	Binary	Clinical database	Supervised	Brain damage	Fivefold
Wang et al., (2013) [18]	Retrospective cross-sectional	647	Develop a strategy to find a minimum number of predictive biomarkers and, eventually, enhance the early identification of baby sepsis.	SVM and Lasso linear Regression	Binary	Clinical database	Supervised	Morbidity and mortality	Not reported
Chaves and Nascimen, (2014) [19]	Retrospective predictive modeling study	92	Create a language model to calculate neonatal mortality risk.	Fuzzy logic model	Not reported	HER	Not reported	Mortality before discharge from hospital	Not reported
Mani et al., (2014) Mani et al., (2014) [20]	Retrospective predictive modeling study	299	Create models using "off-the-shelf" health information to forecast late-onset sepsis and, eventually, lower the neonatal mortality rate.	AODE, RF, Naïve Bayes, and classification and regression trees	Binary	EHR	Supervised	Mortality	Fivefold
Wang et al., (2014) [21]	Retrospective observational study	45	Find diagnostic biomarkers to forecast death in children with severe sepsis, such as angiopoietin-1, angiopoietin-2, and bicarbonate.	SVM	Binary	Clinical database	Supervised	Mortality	Not reported
Kennedy and Turley, (2011) [22]	Retrospective predictive modeling study	212	Create and evaluate cardiac arrest forecasting systems to track improvements in prediction accuracy for young patients who are at risk of death or impairment.	SVM	Multiclass	Research database	Supervised	Morbidity and mortality	10-fold
Toltzis et al., (2015) [23]	Retrospective predictive modeling study	150000	Create a crisis measures of care triage allocation plan to assess children's mortality risk, duration of stay, and ventilation needs.	Linear regression	Regression	Virtual PICU database	Supervised	LOS in PICU, mechanical ventilation, and mortality	Not reported
Campbell et al., (2016) [24]	Retrospective image analysis	Not reported	Create Crisis Guidelines for Care Allocation Determine the requirements for ventilation, duration of stay, and mortality risk in children by using quantitative image analysis to detect the retina's vascular characteristics in order to diagnose severe illness and potential blindness in premature newborns.	iROP	Multiclass	Clinical database	Supervised	The iROP classification of plus disease's percentage accuracy	Not reported
Carlin et al., (2018) [25]	Retrospective predictive modeling study	7256	Estimate each patient's physiologically acceptable condition at PICU discharge to ascertain the overall length of stay.	Recursive neural network	Regression	HER	Supervised	LOS	Not reported
Irles et al., (2018) [26]	Retrospective predictive modeling study	76	Predict intestinal perforation associated with necrotizing enterocolitis and look into factors that could indicate a newborn's neurological decline and mortality.	ANN	Regression	EHR	Supervised	Development of colitis	Not reported
Lamping et al., (2018) [27]	Randomized controlled trial	296	Create and verify a diagnostic model to distinguish between noninfectious systemic immune response syndrome and pneumonia in children using commonly available parameters.	RF	Binary	HER	Supervised	The presence of sepsis or not transmissible systemic inflammatory reaction syndrome.	Three-fold
Shirwaikar et al., (2018) [28]	Retrospective predictive modeling study	Not reported	Utilize classification algorithms to forecast whether caffeine will be sufficient and effective in treating newborns' apneic episodes.	Perceptrons with multiple layers and deep belief networks	Regression	Research database	Supervised	Mortality, medication effectiveness, and apneic episodes	Not reported
Williams et al., (2018) [29]	Retrospective study	Not reported	Examine medical information that could forecast PICU patients' mortality risk, duration of stay, and use of inotropes for ventilation.	k-mean clustering	Not reported	EHR and research database	Unsupervised	LOS, mortality, and the usage of inotropes, ventilation, and intubation	Not reported
Chaichulee et al., (2019) [30]	Retrospective observational study	15	Estimate measurements in NICU patients by using frameworks to identify time periods and skin locations of interest.	CNN	Binary	Video camera	Supervised	Cardiorespiratory signal	Two-fold
Kayhanian et al., (2019) [31]	Retrospective study	94	Determine which admission laboratory factors are associated with children's outcomes following traumatic brain injury.	SVM	Binary	HER	Supervised	Uncertainty surrounds favorable versus bad outcomes; background mortality is stated.	Fivefold
Kim et al., (2019) [32]	Retrospective observational cohort study	1723	Explain the creation and assessment of the Pediatric Risk of Mortality Prediction Tool, which predicts mortality in PICU patients in real time.	CNN	Binary	HER	Supervised	PICU mortality	Fivefold
Masino et al., (2019) [33]	Retrospective case-control design	618	To reduce neonatal mortality, create a model that can identify sepsis at least four hours before clinical detection.	k-NN, LR, AdaBoost, GB, Gaussian process, Naïve Bayes, RF, and SVM	Binary	EHR	Supervised	Mortality	10-fold
Matam et al., (2019) [34]	Retrospective observational cohort design	538	Determine whether an automated examination of multivariate physiological data would lower infant mortality by enabling early detection and prediction of cardiac arrests.	Nonlinear methods for signal processing	Binary	Research database	Supervised	Mortality	Not reported
Moccia et al., (2019) [35]	Experimental observational design	16	Provide a novel method for limb estimation of pose to evaluate health and identify cognitive and motor impairments in premature babies.	CNN	Regression	Video camera	Supervised	Capacity to identify both temporal and spatial characteristics from video recordings of a newborn's limb movements; supposedly intended to evaluate health status and identify cognitive/motor abnormalities early	Not reported
Nagori et al., (2019) [36]	Observational study	Not reported	Create an automated, noninvasive algorithm to forecast shock in kids ages 0-12.	RF and generalized linear model	Regression	Thermal camera	Supervised	Evaluation of model learning, shock index, and hemodynamic shock	Not reported
Ornek et al., (2019) [37]	Observational study	38	To lower neonatal mortality, use infrared imaging to identify health conditions and diagnose illnesses.	CNN	Binary	Thermal camera	Supervised	Early disease and anomaly diagnosis leads to the detection of neonatal health status and a decrease in mortality.	10-fold
Ruiz et al., (2019) [38]	Retrospective observational cohort	93	Reduce the duration of stay, morbidity, and death in infants with single-ventricle physiology younger than six months prior to second-stage surgery by achieving early prediction of crucial events.	Naïve Bayes	Multiclass	HER	Supervised	Capacity to forecast crucial events and patient decline in as little as eight hours; supposedly intended to lower morbidity, mortality, length of stay, and medical expenses	Fivefold
Fraiwan & Alkhodari, (2020) [39]	Retrospective observational cohort	37	Examine how to maximize neonates' healthy brain development by using a short-term, long-term learning method in automatic sleep stage scoring.	Memory neural network	Multiclass	Research database	Supervised	Connected to normal brain development	10-fold
Hamilton et al., (2020) [40]	Prospective study	Not reported	Calculate the risk of serious neonatal morbidity in preterm births with gestations less than 32 weeks, such as mortality, intraventricular hemorrhage, ≥28 days on a ventilator, periventricular leukomalacia, or stage III necrotizing enterocolitis.	Not reported	Not reported	Research database	Not reported	Any one of five outcomes - death, grade 3 or 4 intravenous tricular hemorrhage and ≥28 days on ventilator, periven tricular leukomalacia, or stage III necrotizing enterocolitis - were considered indicators of severe newborn morbidity.	Not reported
Gordon et al., (2020) [41]	Nested case-control study	38	Determine and contrast modified metabolic pathways and metabolites in babies with bacterial meningitis that has been culture-proven.	RF	Binary	EHR	Supervised	Morbidity and mortality	Not reported

The included studies reported varying levels of AI model performance, with some providing comprehensive evaluation metrics while others lacked key performance indicators. Accuracy was explicitly reported in a few studies, ranging from 0.7 [14] to 0.99 [36,37]. AUROC was documented in several studies, with values as high as 0.99 [37], indicating strong predictive capabilities. Sensitivity (Recall) ranged from 0.69 to 1, with Clark et al. [16] achieving perfect recall (1.0), while specificity values varied widely, with some studies reaching 1.0 [11,31]. However, precision and F-measure were frequently underreported, limiting comprehensive performance comparisons. The variation in reported metrics and the absence of consistent benchmarking across studies highlight the need for standardized evaluation frameworks in AI applications within pediatric intensive care (Table 3).

**Table 3:** Variability in AI reporting among included studies CPD: cerebral palsy detection

Study	Accuracy	AUROC	Sensitivity/recall	Specificity	Precision	F-measure
Saria et al., (2010) [10]	Not reported	0.91	0.86	0.96	Not reported	Not reported
Saadah et al., (2014) [11]	Not reported	Not reported	0.82	1	Not reported	Not reported
Matić et al., (2015) [12]	Not reported	0.97	0.95	Not reported	Not reported	Not reported
Caparros-Gonzalez et al., (2018) [13]	Not reported	0.85	0.81	0.76	Not reported	Not reported
He et al., (2018) [14]	0.7	0.76	Not reported	Not reported	Not reported	Not reported
Podda et al., (2018) [15]	Not reported	0.91	Not reported	Not reported	Not reported	Not reported
Clark et al., (2019) [16]	Not reported	Not reported	1	Not reported	1	Not reported
Temko et al., (2011) [17]	Not reported	Not reported	Not reported	Not reported	Not reported	Not reported
Wang et al., (2013) [18]	0.87	Not reported	Not reported	Not reported	Not reported	Not reported
Chaves and Nascimen, (2014) [19]	Not reported	0.81	Not reported	Not reported	Not reported	Not reported
Mani et al., (2014)	Not reported	0.78, 0.77, 0.53, and 0.65	Not reported	Not reported	Not reported	Not reported
Wang et al., (2014) [21]	Not reported	Not reported	Not reported	Not reported	Not reported	Not reported
Kennedy and Turley, (2011) [22]	0.94	0.97	Not reported	Not reported	Not reported	Not reported
Toltzis et al., (2015) [23]	Not reported	0.87	Not reported	Not reported	Not reported	Not reported
Campbell et al., (2016) [24]	0.95	Not reported	Not reported	Not reported	Not reported	Not reported
Carlin et al., (2018) [25]	Not reported	Not reported	Not reported	Not reported	Not reported	Not reported
Irles et al., (2018) [26]	Not reported	Not reported	Not reported	Not reported	Not reported	Not reported
Lamping et al., (2018) [27]	Not reported	0.78	Not reported	Not reported	Not reported	Not reported
Shirwaikar et al., (2018) [28]	0.92	0.91	0.9	0.93	0.94	0.81
Williams et al., (2018) [29]	Not reported	Not reported	Not reported	Not reported	Not reported	Not reported
Chaichulee et al., (2019) [30]	0.94	0.98	0.94	0.94	0.94	Not reported
Kayhanian et al., (2019) [31]	Not reported	Not reported	0.8	0.99	Not reported	Not reported
Kim et al., (2019) [32]	0.84-0.773	0.89-0.97	0.8	0.8	Not reported	Not reported
Masino et al., (2019) [33]	Not reported	0.85, 0.87, 0.85, 0.84, 0.86, and 0.86	Not reported	0.72, 0.74, 0.74, 0.73, 0.74, and 0.72	Not reported	Not reported
Matam et al., (2019) [34]	0.91	Not reported	0.71	0.69	Not reported	Not reported
Moccia et al., (2019) [35]	Not reported	Not reported	0.9	Not reported	Not reported	Not reported
Nagori et al., (2019) [36]	0.75, 0.77, and 0.69 for 0th, third, and 12^th^ hour	0.99 and 0.94 for abdomen and foot segmentation	0.69, 0.72, 0.48, and 0.67 for manually calculated CPD; 0.58, 0.65, 0.58, and 0.62 for automated detection of CPD	0.79, 0.78, 0.81, and 0.68 for manually calculated CPD; 0.81, 0.82, 0.74, and 0.73 for automated detection of CPD	Not reported	Not reported
Ornek et al., (2019) [37]	0.99	0.99	0.99	0.99	Not reported	Not reported
Ruiz et al., (2019) [38]	Not reported	0.88	0.84	0.95	Not reported	Not reported
Fraiwan and Alkhodari, (2020) [39]	0.96	Not reported	0.94	0.97	0.94	0.94
Hamilton et al., (2020) [40]	Not reported	Not reported	Not reported	Not reported	Not reported	Not reported
Gordon et al., (2020) [41]	Not reported	0.97	Not reported	Not reported	Not reported	Not reported

Risk of Bias Assessment

The risk of bias assessment was conducted using an appropriate tool to evaluate potential biases across the included studies. The analysis revealed variability in study quality, with several studies demonstrating a high risk of bias due to incomplete reporting of key performance metrics, such as accuracy, precision, and F-measure. Notably, while studies like Ornek et al. [37] and Chaichulee et al. [30] reported high AUROC values (0.99 and 0.98, respectively), many others lacked comprehensive sensitivity and specificity data, limiting the robustness of their findings. Additionally, studies such as Temko et al. [17], Wang et al. [18], Carlin et al. [25], and Williams et al. [29] failed to report crucial metrics, introducing concerns about reporting bias. The inconsistency in reporting precision and F-measure across multiple studies further complicates the assessment of predictive performance. Overall, while several studies exhibited strong diagnostic capabilities, the absence of standardized reporting across all included studies suggests a moderate to high risk of bias, necessitating cautious interpretation of the findings. The detailed risk of bias assessment is presented in Table 4.

**Table 4:** Risk of bias assessment using PROBAST PROBAST: Prediction model Risk Of Bias Assessment Tool

Study	Participants	Predictors	Outcome	Analysis
Saria et al., (2010) [10]	Low	Unclear	Low	High
Saadah et al., (2014) [11]	Unclear	Unclear	Unclear	Unclear
Matić et al., (2015) [12]	Low	Low	Low	Low
Caparros-Gonzalez et al., (2018) [13]	High	Unclear	Unclear	High
He et al., (2018) [14]	Unclear	High	Unclear	Unclear
Podda et al., (2018) [15]	Unclear	Unclear	Unclear	Unclear
Clark et al., (2019) [16]	High	Low	High	High
Temko et al., (2011) [17]	Unclear	Unclear	Unclear	Unclear
Wang et al., (2013) [18]	Low	High	Low	High
Chaves and Nascimen, (2014) [19]	Unclear	Unclear	Unclear	Unclear
Mani et al., (2014) [20]	Unclear	Unclear	Unclear	Unclear
Wang et al., (2014) [21]	Unclear	Unclear	Unclear	Unclear
Kennedy and Turley, (2011) [22]	Low	Low	Low	Low
Toltzis et al., (2015) [23]	High	High	High	High
Campbell et al., (2016) [24]	Low	Low	Low	Low
Carlin et al., (2018) [25]	Unclear	Unclear	Unclear	Unclear
Irles et al., (2018) [26]	Unclear	Unclear	Unclear	Unclear
Lamping et al., (2018) [27]	Unclear	Unclear	Unclear	Unclear
Shirwaikar et al., (2018) [28]	Low	Low	Low	Low
Williams et al., (2018) [29]	Unclear	Unclear	Unclear	Unclear
Chaichulee et al., (2019) [30]	Low	Low	Low	Low
Kayhanian et al., (2019) [31]	High	Unclear	High	High
Kim et al., (2019) [32]	Low	Low	Low	Low
Masino et al., (2019) [33]	Low	Low	Low	Low
Matam et al., (2019) [34]	High	High	High	High
Moccia et al., (2019) [35]	Unclear	Unclear	Unclear	Unclear
Nagori et al., (2019) [36]	Low	Low	Low	Low
Ornek et al., (2019) [37]	Low	Low	Low	Low
Ruiz et al., (2019) [38]	High	High	High	High
Fraiwan and Alkhodari, (2020) [39]	Low	Low	Low	Low
Hamilton et al., (2020) [40]	Unclear	Unclear	Unclear	Unclear
Gordon et al., (2020) [41]	Low	Low	Low	Low

Discussion

AI has the ability to revolutionize pediatric healthcare by enhancing clinical judgment and streamlining the provision of care [42]. This is especially true in intensive critical care settings where doctors frequently have to make life-saving choices [13]. This systematic review represents one of the first comprehensive efforts to explore the application of AI in improving patient outcomes in PICUs.

Seven studies identified the use of AI at the bedside, and each one was linked to better health outcomes [11,15,16,29,33,36,41]. Furthermore, the studies in this study were divided into several impact emphasis areas and seem to align with the current state of healthcare AI, which includes applications to aid with early disease diagnosis, clinical decision-making, and patient triage.

Clinical Influence and Readiness of AI

The majority of new AI technologies have not yet been embraced by the broader medical community, despite the belief of some experts that AI will transform healthcare in the future. Not all technological advancements result in tools that are suitable for everyday use [16]. While AI has made significant strides in adult medicine, particularly in intensive care and inpatient settings, its integration into pediatric care remains in its early stages [43]. Like other medical tools and therapies, AI applications in healthcare must undergo rigorous testing before being adopted into routine clinical practice [23,25]. Due to concerns about the safety and effectiveness of these instruments and methods in very susceptible groups, such as extremely preterm infants, the application of AI in pediatric critical care might fall behind other medical specialties. Few studies have been conducted on the application of AI in low- to middle-income nations where a large number of newborns and young children need acute care [20,28,30,38]. An essential area for future research is examining the impact of AI on these clinical environments and its implications for patient care and healthcare workflows.

Few of the papers in our evaluation that applied the technology readiness level (TRL) system were deployed in an intensive care unit or in the implementation phase [31,32]. In line with the initial European Commission report, which showed that greater TRLs are possible for AI algorithms that emphasize particular skills, the AI models in these advanced phases were narrowly focused on tasks [40]. The TRL system may offer some understanding into which AI algorithms are advancing into pediatric critical care, as it is challenging to forecast what innovations will become a standard component of daily care in the PICU [25].

Researchers reported AI algorithms that performed better than traditional modalities in the majority of experiments, which may have had an indirect effect on health outcomes. It's unclear if these AI technologies will actually affect patients' health results. To evaluate the effect of AI on outcomes (such as disease burden, length of stay (LOS), readmissions, and death), future AI research must include prospective, experimental study designs in addition to trustworthy, verified metrics and implementation frameworks [19].

AI Performance Metrics and Variability

Due to significant reporting variation, evaluating the ML algorithms in our review was challenging. For example, one study utilized the AUROC quantitative result to gauge the success of the AI model, whereas another study used recall. Even though metrics like AUROC are seen to be better, providing relevant analysis with them necessitates a high level of technical expertise from researchers [36]. While it makes sense for certain AI models to employ distinct performance indicators, this degree of reporting variety complicates the comparison and assessment of AI systems. Furthermore, variations in the algorithms employed, the training data, and the absence of geographical or spectrum validation all hinder the application of these techniques in clinical practice [35]. When examining information collected from the same population of interest and evaluating AI performance overall using metrics, AI models must be similar. Otherwise, inaccurate AI algorithms used to make medical decisions may have a negative impact on patient outcomes [10].

Challenges and Future Directions in AI for Pediatric Intensive Care

For AI technologies to serve as efficient decision support systems and assist intensivists in real time, they must be able to interpret and decode gigabytes of clinical data [11]. Interestingly, even though the review's topic is critical care, several of these issues might be common to pediatric inpatient settings. The fact that the hospitalized patients' data required for interpretation and training is usually complex, varied among institutions, and primarily unstructured presents a major obstacle to the application of AI technologies. Digital data is not routinely collected or precisely measured and documented, despite the fact that it is now easier to access for analysis [13].

Natural language processing (NLP) is required for unstructured text data, such as clinical notes in the majority of inpatient settings [44]. Unfortunately, it takes a lot of money and effort to annotate big text passages in order to train any model. Only one study in our evaluation used NLP, indicating that this technology is still relatively new to AI developers working in neonatal and pediatric intensive care settings [15].

Unstructured data can be difficult to organize, annotate, and analyze [45]. Researchers have just recently been able to create AI-based methods for obtaining unstructured information from the electronic medical record (EMR). Clinicians rely on unprocessed EMR data to comprehend intricate illness processes, which presents a challenge for implementing AI in hospital-based practice [46]. Furthermore, AI models that are trained on unstructured and possibly biased input data may produce biased and deceptive outputs that have a detrimental impact on patient care. Our review's studies don't address particular methods for dealing with skewed or unbalanced data, nor do they assess for bias in the data itself, which makes the conclusions less reproducible.

Structured data-trained algorithms can guarantee the extraction of high-quality data, enhance AI performance, and make AI input easier to utilize. Future research could examine the idea of "representation learning." In representational learning, AI models generate an abstract representation of each patient's data for medical record extraction by automatically learning features and using a predictive model. In addition to technical constraints, a number of human factors and system-integration hurdles may make it more difficult to integrate AI into newborn and pediatric critical care. Future research should examine how AI affects clinical workflow and concentrate on the elements that affect clinician faith in AI. Over time, providers may develop cognitive biases as a result of using AI. Clinicians might, for example, start to blindly accept the data produced by the AI system due to its reliable and incredibly effective performance, or they might reject AI output data without fully taking into account how it may affect outcomes due to prior experiences. From a quantitative standpoint, AI that has been trained on a certain patient subset may be biased and generate findings that are valid exclusively for that patient group. Future researchers will need to enlist the assistance of medical professionals, computer scientists, and human factors engineers to overcome such challenges.

AI algorithms frequently fail to understand the intricacies and comorbidities inherent in pediatric inpatient treatment, in addition to the challenges associated with AI and data collection from the EMR. For optimal performance, AI algorithms make use of all viable and accessible signals. However, the inclusion of unreliable confounders could hinder the algorithm's capacity to generalize to new data sets. Understanding the input data that an AI has been trained on and compared against is essential. The notion that all doctors are the best may not always be accurate in research when algorithms and doctors were compared [47].

A retrospective study approach was used in a number of studies, which means that algorithms (supervised learning) were trained and tested using previously labeled data [12,23,31]. Importantly, since the accuracy of AI is likely to deteriorate when faced with real-world data that varies from algorithm training, its usefulness can only be recognized through future studies [20,48].

Lastly, the majority of studies reduced pediatric complications to binary categorization types, classifying a person into one of two groups (illness vs. no illness) based on a number of variables [27,35,39]. However, this method ignores the possibility that a patient may have several comorbidities, each with varying degrees of severity or interdependency. We did not provide a summary or analysis of these elements in our study because the approach of these studies rarely addresses the obstacles to adopting AI. This is yet another crucial subject that needs more studies.

Limitations

To evaluate the impact of AI on health outcomes, we utilized the EIT Health (European Institute of Innovation and Technology Health) joint report, which was developed with adult healthcare settings in mind. In most of the focus areas, this categorization system remained applicable to our patient population. However, we excluded non-English articles, which may have limited our ability to capture AI applications in a broader, more diverse population. Additionally, our search was restricted to the past 10 years, meaning that earlier relevant studies may not have been included. While this timeframe was chosen to ensure relevance to contemporary AI developments, it is possible that some foundational studies were missed. The non-retrieval of such studies may introduce a degree of selection bias, particularly if older studies contained insights into AI methodologies still applicable today. Lastly, the heterogeneity in research methods and outcome measures prevented us from conducting a meta-analysis, further limiting the generalizability of our findings.

## Conclusions

Although there is a growing body of research that promotes the use of AI to enhance pediatric health outcomes, it will take some time before AI becomes widely implemented into routine pediatric healthcare practice. For example, using supervised AI algorithms for inpatient data presents challenges because input training on trained models does not allow for efficient utilization of AI in real-world scenarios (such as complex critical illnesses with comorbidities), and AI typically struggles to process unsupervised information for clinical data extraction. Further research is needed to fully realize the potential advantages of AI in pediatric healthcare facilities.
